# Marine Arthropods as a Source of Antimicrobial Peptides

**DOI:** 10.3390/md20080501

**Published:** 2022-08-02

**Authors:** Juan Pablo Saucedo-Vázquez, Fernando Gushque, Nelson Santiago Vispo, Jenny Rodriguez, Marco Esteban Gudiño-Gomezjurado, Fernando Albericio, Markus P. Tellkamp, Frank Alexis

**Affiliations:** 1 CATS Research Group, School of Chemical Sciences & Engineering, Yachay Tech University, Hda. San José s/n y Proyecto Yachay, Urcuquí 100119, Ecuador; jsaucedo@yachaytech.edu.ec; 2 School of Biological Sciences & Engineering, Yachay Tech University, Hda. San José s/n y Proyecto Yachay, Urcuquí 100119, Ecuador; egushque@yachaytech.edu.ec (F.G.); nvispo@yachaytech.edu.ec (N.S.V.); 3 Escuela Superior Politécnica del Litoral (ESPOL), Centro Nacional de Acuicultura e Investigaciones Marinas (CENAIM), Campus Gustavo Galindo Km 30.5 Vía Perimetral, Guayaquil 090211, Ecuador; jenrodri@espol.edu.ec; 4 Facultad de Ciencias de la Vida (FCV), Escuela Superior Politécnica del Litoral, ESPOL, Guayaquil 090708, Ecuador; 5 School of Chemistry and Physics, University of KwaZulu-Natal, Durban 4001, South Africa; albericio@ukzn.ac.za; 6 Networking Centre on Bioengineering, Biomaterials and Nanomedicine (CIBER-BBN), Department of Organic Chemistry, University of Barcelona, 08028 Barcelona, Spain; 7 Institute for Advanced Chemistry of Catalonia (IQAC-CSIC), 08034 Barcelona, Spain; 8 Politecnico, Universidad San Francisco de Quito USFQ, Quito 170901, Ecuador

**Keywords:** antimicrobial peptides, AMP, crustaceans, defense system, drug, shrimp

## Abstract

Peptide therapeutics play a key role in the development of new medical treatments. The traditional focus on endogenous peptides has shifted from first discovering other natural sources of these molecules, to later synthesizing those with unique bioactivities. This review provides concise information concerning antimicrobial peptides derived from marine crustaceans for the development of new therapeutics. Marine arthropods do not have an adaptive immune system, and therefore, they depend on the innate immune system to eliminate pathogens. In this context, antimicrobial peptides (AMPs) with unique characteristics are a pivotal part of the defense systems of these organisms. This review covers topics such as the diversity and distribution of peptides in marine arthropods (crustacea and chelicerata), with a focus on penaeid shrimps. The following aspects are covered: the defense system; classes of AMPs; molecular characteristics of AMPs; AMP synthesis; the role of penaeidins, anti-lipopolysaccharide factors, crustins, and stylicins against microorganisms; and the use of AMPs as therapeutic drugs. This review seeks to provide a useful compilation of the most recent information regarding AMPs from marine crustaceans, and describes the future potential applications of these molecules.

## 1. Introduction

Bioactive peptides have a broad spectrum of biological activities; from these, their immune signaling role stands out, due to their high selectivity and specificity for extracellular and intracellular target receptors. They are found in all living systems and play a decisive role in several physiological functions [1]. Given their vast variability and bioactive properties, peptides are considered to be pharmacologically ideal, in terms of safety, tolerability, and efficacy profiles, for the design of novel therapeutics for humans [2], thus increasing the wide family of traditional bioactive natural products from diverse sources such as plants, animals, and even bacteria [3,4]. Regulatory agencies have already approved around 100 pharmaceuticals in which a peptide serves as the active pharmaceutical ingredient (API) [5,6]. Of note, although cancer was traditionally the most important target for peptide-based drugs, recent years have witnessed the presence of bioactive peptides in practically all targets, with a special incidence in metabolic diseases and in radiopharmaceuticals [7].

Bioactive peptides can be isolated from natural sources. Such is the case of insulin, adrenocorticotropic hormone (ACTH), and calcitonin (canine or bovine pancreas, bovine or porcine pituitary glands, and salmon ultimobranchial gland), or they can be chemically synthesized or produced via fermentation [8]. In that regard, venoms from a wide range of animals, including cnidarians, echinoderms, mollusks, arthropods, and vertebrates, are now recognized sources of bioactive peptides for new potential therapeutics. In most cases, the isolation of peptides from natural sources is associated with the drug discovery process, which seeks to identify peptide sequences with the potential to become peptide drugs that can be produced synthetically or through genetic engineering [9]. Given their lack of stability in plasma and their poor ability to be transported through cell membranes as a result of the hydrophilicity inherent to their structures, native peptides face some limitations to becoming drugs. In this regard, the field of peptide chemistry is critical for the development of stable, active, and specific sequences of peptides with potential therapeutic uses. To this end, current strategies include the use of D-enantiomers, the modification of side chain length, the use of N-methylation or bulky residues, terminal capping, and cyclization to overcome protease degradation.

In addition, peptides are considered suitable templates for introducing chemical modifications, resulting in peptide mimetics that have also been approved by the corresponding regulatory agencies. Heterologous peptide discovery through synthetic library screening has resulted in the approval of only a few peptide drugs for clinical use. However, peptide analogues show improved properties over native peptides and they are the major chemical basis for new peptide drugs. In conclusion, advances in peptide chemistry and synthesis, and in drug delivery systems have brought about the success of peptide therapeutics and their conjugates. 

Thus, the use of peptides with biological activity in therapeutics has growth significantly [10,11,12,13,14,15]. In this context, marine organisms are a major source of such molecules because their immune systems exert powerful action against several viral and bacterial diseases [16,17,18]. In this context, antimicrobial peptides (AMPs) play a key role in strengthening the immune systems of these creatures. The relevance of bioactive peptides is reflected in several reviews on AMPs in mammals [19], amphibians [20], fishes [21], plants [22], insects [23], echinoderms [24], marine arthropods [16,25,26], and penaeid shrimps [27,28]. Remarkable research has been conducted in this area [29]; therefore, our review emphasizes the synthetic chemistry of AMPs and their tentative medicinal applications. We describe recent advances regarding the molecular analysis of crustacean AMPs, concentrating on the antimicrobial mechanisms of these molecules against most pathogens in aquatic arthropods. These peptides not only serve to protect the organism against microbes (including Gram-positive and Gram-negative bacteria, yeast, and fungi) but also have antitumoral effects and mitogenic and immunoregulatory activity [30]. 

The immune response of crustaceans and chelicerates (Ecdysozoa, Arthropoda) is regulated on the external cuticle and the innate immune system, involving humoral and cellular effectors. Hemocytes are essential for the cellular immunity of these organisms, as they are involved in the storage and release of several defense molecules, including AMPs. Around 10% of animal-derived AMPs are from crustaceans, most of them (14 out of 15 families) having been identified in the decapod order (crabs, lobsters, crayfish, and several shrimp species) [31]. In marine crustaceans, the internal defenses rely on cellular and humoral responses (including pattern-recognition receptors/proteins, the production of reactive oxygen species, enzymatic cascades, clotting proteins, and AMPs) of the innate immune system that are mediated by blood cells or hemocytes [32]. The broad spectrum of biological activity shown by AMPs is a result of the wide variability of the subgroups and isoforms in a few families of these bioactive molecules. Indeed, current knowledge with regard to AMPs derived from marine crustaceans refers mostly to Decapoda, and thus does not represent the high diversity of sources [31,33]. AMPs are defined as cationic, amphipathic, and single gene-encoded molecules (also expanded to anionic and multigene-encoded). Their structure predisposes them toward interaction with the bacterial membrane, and they are therefore a promising source of next-generation antibiotics. 

In terms of amino acid composition, AMPs are classified into four groups: single-domain linear α-helical AMPs (e.g., armadillidin and homarin); single-domain AMPs containing Cys residues engaged in disulfide bonds (e.g., defensins, scygonadins, and anti-lipopolysaccharide factors (Alefs)); multi-domain or chimeric AMPs (e.g., penaedins, crustins, hyastatin, etc.); and unconventional AMPs, including multifunctional proteins and protein-derived fragments (e.g., histones and hemocyanin) [31]. Most AMPs display a broad spectrum of antimicrobial properties against bacteria, viruses, fungi, and protozoa. Penaedins and ALFs have potential uses in therapeutics due to a lower risk of resistance, a low toxicity, and potential immune modulator function. A major advantage of AMPs from marine crustaceans is their stability in the gastrointestinal tract environment. 

This review provides a general summary of information from the last five years on the diversity and distribution of AMPs in marine arthropods (crustacea and chelicerata), with a focus on penaeid shrimp (Crustacea, order Decapoda, family Penaeidae). Since all immune molecules in crustaceans, including AMPs, are produced and stored in hemocyte granules [34], we cover the shrimp’s defense mechanism [28,35]. Furthermore, given the complexity and diversity of AMPs, we address the classes of AMPs, and their chemical structures, properties, functions, and chemical syntheses. Additionally, we review the potential uses of AMPs and the inspired synthetic drugs for therapeutic applications. Finally, we include a section on the future perspectives for AMPs in the context of health.

## 2. Bioactive Peptides in the Subphylum Crustacea

### Diversity and Distribution

AMPs are ubiquitous defense molecules found in all life forms; unicellular organisms are thought to employ them during interspecific interactions, whereas multicellular life forms use them as part of their innate immune systems [36]. Despite the economic importance of many crustaceans, AMPs have not been extensively studied in these organisms, and research efforts continue to be channeled into identifying novel AMPs in a range of crustacean species [37]. Such efforts are likely to lead to the discovery of new AMPs, which will help to elucidate patterns in the evolution of these peptides in this animal group. To date, six AMPs have been identified in shrimp (order Decapoda, family Penaeidae) and 16 chemically distinct AMPs within the subphylum Crustacea (Table 1). 

Interestingly, not all of the AMPs are found in all the crustacean groups. While some are found in all Crustacean orders [46], hinting at an ancient origin of these molecules, others are limited to one or a few families, thereby suggesting a more recent evolution (Table 1). A good example of an ancient class of AMPs are the ALFs, which have undergone intense diversification. In contrast, penaeidins are a class of AMPs found in a single family of crustaceans, where they have diversified into four distinct subtypes. Therefore, there is a vast diversity of AMPs that could be elucidated through future bioinformatics and proteomic searches [59]. Clues as to the causes of the great diversification of some AMPs in the Crustacea subphylum can be found by looking at the amino acid composition. Many AMPs show a clear amino acid bias, being rich in Pro, Pro/Arg, or Gly. These amino acids cause the peptide to acquire certain structures and have a cationic nature (although a few anionic AMPs do exist). These properties predispose the peptides to interact in certain ways with the membrane of pathogens. 

Thus, some AMPs may have resulted from nonsense mutations in large proteins that have domains rich in these amino acids. Pro-rich proteins are ubiquitous in eukaryotic genomes, and are thus common signaling molecules. In general, Pro/Arg-rich proteins that can provide a blueprint for AMPs are not as common, but they can be found throughout the genomes of several eukaryotic organisms and have diverse functions, such as C9ORF72 [60]. However, within the Crustacea subphylum, Pro-rich AMPs have a diverse evolutionary origin [61]. Gly-rich proteins are common throughout Eukaryotes and often have diverse functions [62]. Prior to the mutations that may have led to the creation of putative “pre-AMPs”, the gene in question would have had to undergo duplication to avoid the loss of functionality. These genes are molecular “hopeful monsters”, resulting in new lineages of selfish genes that confer an important immune advantage to the individual, vis a vis population, in response to pathogens.

The proposed model for the diversification of other AMPs would also hold for the truncation of proteins that preserve entire domains, such as the single whey acidic protein (WAP) which contains eight well-conserved cysteine residues forming a four-disulfide core [31]. Truncation of proteins is followed by a nonsense mutation in one or more orthologs, of which only those with antibacterial properties confer an advantage to the animal. These novel AMPs may then have further multiplied through gene duplication and mutation. The observation that the antibacterial activity of AMPs is due to the properties of a certain group of amino acids suggests that the de novo synthesis of artificial AMPs could stem from the search of amino acid sequences with a similar bias or the search for motifs or domains with a heavy bias in the aforementioned amino acids [59].

## 3. Defense Mechanisms in Crustaceans

### 3.1. Hemocytes

Crustaceans belong to the phylum Arthropoda, and despite not having the sophistication of an adaptive immune system, they have a highly effective innate immune system to fight infections [63]. The first lines of crustacean defense are internal and external physical barriers. A strong external cuticle protects the body and the stomach, and the peritrophic membrane covers the midgut and hindgut [64]. The circulatory system of crustaceans is open; thus, the circulatory fluid, called hemolymph, flows freely through the circulatory system, which has open endings into the lacunal system (the hemocoel), which is in direct contact with tissues. The hemolymph has two components: cellular and humoral. The former is composed of hemocytes, the main immune effectors of the crustacean immune system. In contrast, several proteins, namely the respiratory pigment hemocyanin, the clotting factor, and several AMPs, among others, make up the humoral component. The crustacean hemolymph performs the functions of both the lymph and blood, and thus its biochemical and cellular components reflect the dual function of this tissue. For example, hemocyanin transports oxygen and participles in the immune response [65,66,67].

The innate immunity of crustaceans consists of a cellular and humoral response. The former involves the hemocytes, classified into three major types: hyaline cells (HCs), large granular cells (LGCs), and small-granular cells (SGCs). These cells exert diverse roles (Wu et al., 2019) as they interact with pathogens. HCs are involved in phagocytosis and coagulation, while SGCs participate in phagocytosis, nodule and capsule formation, and the release of the molecules of the prophenoloxidase (proPO) system and AMPs. LGCs store immune effector molecules, AMPs, and enzymes of proPO [68,69,70]. The proportions of each type of hemocyte in the hemolymph depend on various factors, such as molting [71,72]. Hemocytes are produced by hemopoietic tissues. In shrimp, these tissues are located as various epigastric lobules, mainly latero-dorsally to the cardiac stomach and in the proximal region of the maxillipeds. Hemopoietic hemocyte growth and differentiation is likely to be mediated by cytokines [68,73,74].

### 3.2. Cellular Reactions

Given the open nature of the shrimp circulatory system, hemocytes easily infiltrate tissues, and can respond in a localized way or migrate to specific tissues (gills, lymphoid organs, and connective tissue), where they trigger cellular reactions or release a range of compounds. The cellular reaction includes phagocytosis, which is performed mainly by HCs and SGCs, and is accompanied by degradative reactions via the generation of lytic enzymes or reactive oxygen or nitrogen species (ROS and RNS) generated during the respiratory burst [75]. In response to massive invasions of microorganisms or large microorganisms, hemocytes form cell aggregations called nodules. Denser cell aggregations made up of several layers of hemocytes are known as capsules. Sometimes, nodules or tubules of the hepatopancreas (invaded by *Vibrio* spp.) appear to be encapsulated by hemocytes. Melanin, a sticky and toxic dark pigment, is deposited in a very localized way in the central areas of nodules and capsules, or in wounds during the healing process. Melanin is generated by phenoloxidase, an enzyme that is produced via the activation of proPO system. The proPO system, one of the main immunoeffector responses of crustaceans, comprises a series of enzymatic cascade reactions [76] triggered by components of the cell walls of microorganisms, such as β-1,3-glucans from fungi, or lipopolysaccharide (LPS) and peptidoglycans from Gram-negative and Gram-positive bacteria, respectively [77]. Melanin formation generates intermediate reactive compounds (quinones and ROIS), which are more toxic than melanin. ProPO oxidase is regulated by several protease inhibitors, among them, pacifastina and α2-macroglobulin, produced by HCs. α2-macroglobulin inhibits serine proteases of the proPO cascade, and possibly pathogen proteases [78]. Hemocytes also participate in another cellular reaction, namely, extracellular traps (ETs). This process involves the formation of DNA structures and AMPs to trap microorganisms. In the shrimp *Marsupenaeus japonicus*, ETs associated with c-type lysozyme have been described [79].

### 3.3. Other Immune Tissues

In addition to hemocytes, other tissues of the penaeid shrimp are involved in the immune response, including the heart reserve of phagocytes the podocytes of the gills, antennal gland, and the lymphoid organ (LO). The latter, which is near the hepatopancreas, receives hemolymph from the subgastric artery that forms a vascular plexus within this structure. The LO appears to filter viruses from the hemolymph [80]. Very strong hemocyte infiltration is detected in the LO, contributing to the immune functions of this organ. Many genes and molecules associated with hemocytes have been reported in the LO [80,81]. In this regard, a strong signal of penaeidin and α2-macroglobulin has been detected in the edges of tubules of the LO, in the same zone that participates in virus filtration. During infection, mainly that of viral etiology, nodular hyperplasia, known as LO spheroids (LOS) appear in the LO. These hyperplasic cells show a positive signal toward penaeidin and α2-macroglobulin [80]. It has been outlined that LOS are involved in the phagocytosis and destruction of foreign particles [82]. Terminal type C LOS exhibits an apoptosis signal, thus indicating the fate of engulfed material. Ectopic spheroids have also been detected in the connective tissues of other organs (stomach and buccal appendages). 

Initially, there was some controversy regarding whether apoptosis as a response to viral infections in shrimp was a defense mechanism or a pathogenicity mechanism triggered by the virus [83]. The literature has reported that in the early stages of white spot syndrome virus (WSSV) infection, the virus exerts a mechanism to prevent host cells from entering apoptosis [84]. This observation thus indicates that apoptosis is a mechanism associated with the host’s immune response.

### 3.4. Humoral Response

Humoral effectors involve the synthesis and secretion of immune proteins such as AMPs from hemocytes into the hemocoel. Part of the humoral response is the clotting system, which is stimulated by injuries and by the binding of microbial stimulants, such as β-1,3-D-glucans, to receptors on hemocytes. These cells release Ca^2+^-dependent transglutaminases, which promote the polymerization of clotting proteins [85]. Finally, proteins derived from hemocyanin and hemocyanin-derived proteins have multiple functions in the hemolymph, from oxygen transport, antimicrobial activity, and agglutination, to playing a major role in the proPO system [65,66,67,85]. The multiple roles of hemocyanin-related proteins, as well as the diversity of AMP families, suggest that gene duplication and subsequent functional diversification are an important feature of the crustacean immune response. The open nature of the circulatory system of crustaceans, which is composed of vessel systems and a system of lacunae and sinuses surrounding the most important organs [86], facilitates the rapid migration and infiltration of hemocytes in different tissues, where they can then release their molecules in a localized manner.

### 3.5. Recognition of Foreign Molecules

Recognition molecules, including various types of lectins, galectins, β-1,3-glucanase-related protein, Toll-like receptors (TLR), Thioester-containing proteins (TEPs), scavenger receptors, Down syndrome adhesion molecules, and fibrinogen-related proteins, can circulate freely in the hemolymph or be bound to cell membranes. These molecules are able bind to pathogen-associated molecular patterns (PAMPs). Thus, signaling cascades in hemocytes are triggered, leading to the production and exocytosis of AMPs [87] and lysozymes, as well the activation of the proPO system.

### 3.6. Chemical Structure, Properties and Function of AMPs

Most shrimp AMPs are cationic and hydrophobic, as observed for AMPs extracted from other phyla. However, a special class of AMPs are ALFs, in which anionic peptides are common. Due to the structural differences and the phylogenetic origins of the AMPs present in shrimp, they have been divided into several groups, among which the penaeidins, crustins, ALFs, stylicins, and some hemocyanin-derived molecules stand out. The following sections present a description of the main structural characteristics of each of these groups. 

#### 3.6.1. Penaeidins

##### Structure

Penaeidins are a unique family of AMPs that owe their name to the family Penaeidae, since the first penaeidin was isolated from the shrimp *Penaeus vannamei*. Most structural characteristics of penaeidins have been elucidated from peptides extracted from this animal [88]. Penaeidins have been isolated from several species of the Penaeidae family, including *P. setiferus* [89], *M. japonicus* [90], *F. merguiensis* [91], and *M. monoceros* [92], among others. 

Like most AMPs, penaeidins are cationic molecules with isoelectric points that are in the range of 9.34 to 9.84 [16,93]. Their molecular weights (MWs) range from 4.7 to 7.2 kDa, and they are composed of an N-terminus Pro/Arg-rich domain and a C-terminus domain that contains an amphipathic helix with the presence of three disulfide bonds. Such a multi-domain or chimeric structure is unique as compared with the AMPs of other kingdoms.

Since the first description of a penaeidin in 1997 [94], several studies have addressed these bioactive peptides [54,90,92,95,96,97]. Most penaeidins show a highly conserved structure in which the N-terminus Pro-rich domain is the main characteristic. This domain has been observed in other crustacean AMPs, such as in SpPR-AMP1 from *Scylla paramamosain* [98] and the penaeidins of the shrimps *F. indicus* and *M. monoceros* [92]. The Pro-rich domain of the peptide is a determinant of its antimicrobial activity, and in this case, the antibacterial mechanism operates via a non-lytic mechanism in which this domain interacts with the 70S ribosome and disrupts protein synthesis [99].

**Figure 1:**
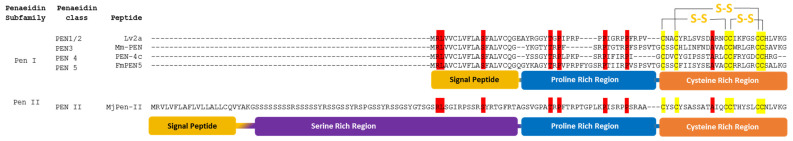
Alignment of five representative penaeidins belonging to subfamilies I and II, showing the conserved Cys-rich region with the characteristic six Cys residues forming three disulfide bonds. Cys residues of the disulfide loop are highlighted in yellow and highly conserved residues, in red.

On the other hand, the C-terminus region holds a Cys-rich domain, in which it has been determined that six of these Cys residues form intramolecular disulfide bridges [100]. The Cys-rich domain is more conserved than the Pro-rich one, and the latter has a variable length depending on the type of penaeidin. Interestingly, penaeidins are structurally related to other Pro-rich peptides, such as apidaecins [101] and drosocins [102]. An alignment of the five classes of penaeidins belonging to the two subfamilies shows such structural signatures (Figure 1).

The classic penaeidins are divided initially into five classes, and then into four larger classes (PEN 1/2 to 5) that share the characteristic Pro-rich and Cys-rich domains, but with some differences that give each of them special biochemical features. PEN1 and PEN2 are grouped into the same class (PEN2), since the amino acid sequence of the former is indistinguishable from that of the latter [103]. The PEN2 class contains peptides with molecular weights (MW) of between 5.5 and 6.6 kDa. Relevant examples of PEN1/2 are Lv2a (Y14925), Lv2b (AF390146), and Lv2c from *P. vannamei,* and Ls2d (AY039205) from *P. setiferus* [16].

**Figure 2:**
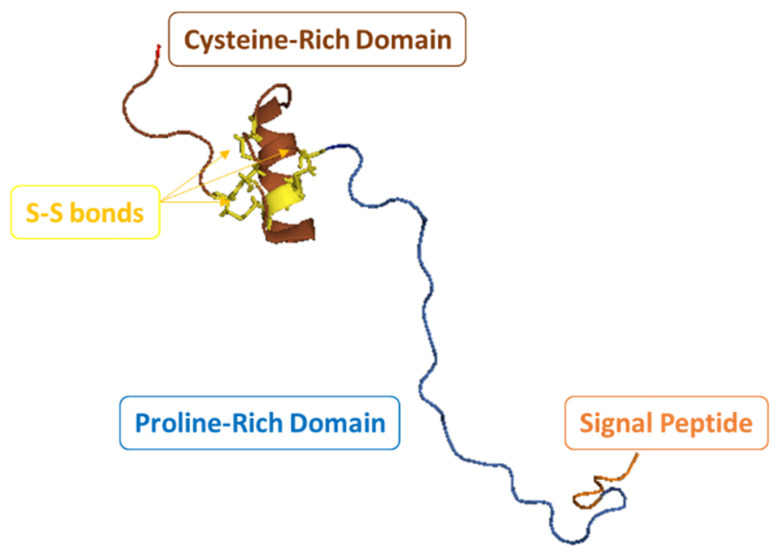
Crystal structure of LVPen3 (PDB 1UEO) from *P. vannamei*, showing the typical amphipathic α−helix structure with three disulfide loops, and the cysteine and proline-rich sequences. Model built with Pymol V1.74.

The PEN3 class contains a particular functional site composed by the triad Val–Tyr–Lys, which serves for adhesion reactions or intermolecular cross-links involved in cell wall assembly. In 2015, a newly identified penaeidin isoform of the peptide from the hemocytes of the Indian white shrimp, *F. indicus* (*Fi*PEN, JX657680), was reported. *Fi*PEN showed a maximum sequence similarity of 63% to a PEN-3 isoform of *P. monodon* [92]. In the same work, the first penaeidin from *M. monoceros* was reported. Named *Mm*PEN (KF275674), this peptide consists of an open reading frame of 338 bp encoding 71 amino acids, and together with *Fi*PEN, it shows the typical characteristics of the PEN3 class, namely 19 amino acid residues in the highly conserved signal peptide region (MRLVVCLVFLASFALVCQG), 24 amino acid residues in the Pro-rich domain, and 28 amino acid residues in the Cys-rich domain. Although many studies have been devoted to penaeidins, only two crystal structures have been resolved to date [89,104]. Of these, the structure of LVPen3 serves as a good model to study other penaeidin molecules [104] (Figure 2).

In 2002, Cuthbertson and co-workers were the first to propose a PEN4 class [103]. Alignments revealed that PEN4 had significantly shorter Pro-rich and Cys-rich domains, resulting in 47-residue peptides instead of the 63-residue peptides of the PEN3 class, which has the longest sequence among penaeidins. The novel PEN4 class was found to be present in both *P. vannamei* (*Lv4a*, AF390147; *Lv4c*, AF390149) and *P. setiferus* (*Ls4d*, AY039207) [103]; PEN3 and PEN4 classes were shown to differ in length and primary sequence, and they are the most diverged representatives of this peptide family in *Penaeus* spp. In 2020, Machado et al., deposited the PEN4 structure of *P. paulensis*. Unfortunately, the characterization of this molecule has not yet been published. However, from a BLAST exercise in the NCBI platform, we found 86% sequence similarity with PEN4a (AF390147) and PEN4c (AF390149) from *P. vannamei* [103], 87% with PEN4-1 (AAX58699) from *P. schmitti* [105], and 85.5% with PEN4-3 of *P. vannamei* (ABA63168) [106].

Recently, a peptide from both the PEN5 and PEN3 classes was cloned and identified from the banana shrimp (*F. merguiensis*) [91]. From the alignment sequence analysis, the authors reported that *Fm*PEN3 (MK092338) and *Fm*PEN5 (MK092339) have 41–67% sequence similarity to the penaeidins from other shrimps. Specifically, *Fm*PEN3 has 64% sequence similarity with PEN3b (ACQ66007) of *P. monodon* [28] and *PEN3-1* (AAP33450) of *F. chinensis*, and 63% with PEN3-2 (ABC33920) of *F. chinensis* [107]. In the same way, *Fm*PEN5 shares 67% sequence identity with PEN5-1 (AAZ79334) and PEN5-2 (AAZ80041) of *F. chinensis* [108]. 

**Figure 3:**
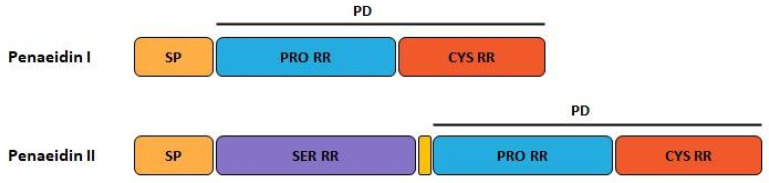
Domains of the classic Penaeidin-I subfamily and the newest Penaeidin-II subfamily; SP = signal peptides, Cys RD = cysteine-rich domain, Prol RD = proline-rich domain, Ser RD = serine-rich domain. Adapted from [90].

Five years ago, a new group of penaeidins was proposed by An et al. [90]. Found in the kuruma shrimp *M. japonicas;* this group was characterized by the presence of a Ser-rich region (Figure 3). This novel structural finding differentiates the four classic classes of penaeidins (PEN 1/2-5) reported previously, and allows for the creation of the new PEN subfamily II. Although this family preserves the characteristic Pro- and Cys-rich regions, the new Ser region appears to confer novel antimicrobial and phagocytic capacity. The new penaeidin identified in *M. japonicus, Mj*Pen-II (AMH87234), was assigned to PEN subfamily II. *Mj*Pen-II was found to be expressed in hemocytes, heart, hepatopancreas, gills, stomach, and intestine, and was upregulated after bacterial challenge.

**Figure 4:**
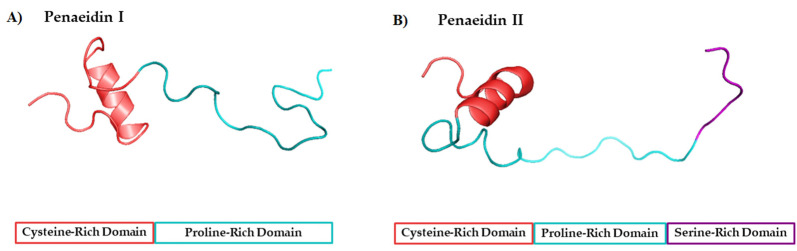
(**A**) Structure of the classic penaeidins (PEN4c), showing conserved Pro-rich (blue) and Cys-rich (red) domains (model obtained with Swiss model); (**B**) New penaeidin (*Mj*Pen-II) family with the same conserved regions and the Ser-rich domain, proposed by An et al. [90].

The full length *MjPen-II* is 417 bp, encoding a 138-amino acid protein that contains a signaling peptide, a Ser-rich region, and a penaeidin domain, the latter including a Pro-rich region and a Cys-rich region (Figure 4). The full-length *Mj*Pen-II has a molecular mass of 14 kDa.

##### Function and Role against Microorganisms

The antibacterial activity of penaedins has been widely described, e.g., recombinant MjPenII (rMjPen-II) shows inhibitory activity against *Bacillus megaterium*, *B. subtilis*, *Escherichia coli*, *Klebsiella pneumoniae*, *Pseudomonas aeruginosa*, *Staphylococcus aureus*, and *Vibrio anguillarum* [90]. Similarly, *Fm*PEN3 and *Fm*PEN5 purified from *P. merguiensis* show antibacterial activity against *Micrococcus lysodeikticus* and *V. parahaemolyticus.* Thus, the minimal inhibitory concentration (MIC_50_) values of FmPEN3 against *V. parahaemolyticus* and *M. lysodeikticus* are >50 μM and 6.25 μM, respectively. On the other hand, the MIC_50_ values of FmPEN5 for *V. parahaemolyticus* and *M. lysodeikticus* are 50 μM and 12.5 μM, respectively [91]. These data suggest that, to trigger an effective immune response against an infectious microorganism, shrimps have developed complex defensive mechanisms based on penaedins.

Although the antibacterial activity of penaedins has been widely described, these molecules have shown an antiviral effect. For instance, in 2018, Xiao et al., found the possible antiviral role of four penaedins from *P. vannamei* during their interaction with structural proteins of the WSSV [35]. Similarly, in 2019, Xiao et al., reported the capacity of PEN2 to bind competitively to the envelope protein VP24 of WSSV, thus interfering with the viral infection process of this crustacean [109].

Findings regarding the structure–function relationship of penaeidins continue to grow. In this regard, more than 40 penaeidins have been reported in several databases, such as APD (aps.unmc.edu/AP/, accessed on 1 March 2021), which contains more than 3200 AMPs from bacteria, archaea, protists, fungi, plants, and animals [110]. Some penaeidin representatives of each subfamily are listed in Table 2.

#### 3.6.2. Crustins

##### Structure

Crustins are a family of AMPs. The first crustin (carcinin) was isolated from the hemocytes of the shore crab, *Carcinus maenas* [111]. Most of the structural characteristics of crustins have been elucidated from *P. monodon* [112] and *P. vannamei* shrimp [48]. However, other peptides belonging to this family have been isolated from other organisms, including *P. setiferus* [113,114], *P. schmitti* [115], and *P. japonicus* [116].

**Figure 5:**
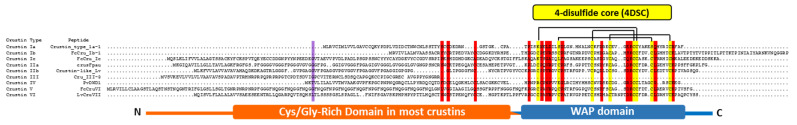
Alignment of nine representative crustins from type I to type VII showing the conserved WAP domain with the characteristic eight Cys residues forming the four-disulfide core (4DSC). Cys residues of the disulfide core are highlighted in yellow and highly conserved residues, in red.

Crustins are cationic Cys-rich AMPs that have a more complex structure than that of penaeidins. Crustins have isoelectric point ranges from 4 to 8, and a MW of between 6 and 22 kDa. These peptides are structurally characterized by Gly-rich, Cys-rich, or Pro-rich regions at the N-terminus, and a WAP domain at the C-terminus (Figure 5). The WAP domain is observed in a large group of peptides with antimicrobial activity, and is strongly associated with such activity. The WAP domain comprises two four-disulfide cores (4DSCs), and together with an additional four conserved Cys residues of the Cys-rich domain, it forms the 12-sulfur core considered as the distinctive feature of type I, II, V, and VII crustins [117]. In the particular case of shrimp crustins, the presence of the extra Gly-rich domain mentioned above appears to differentiate them from the crustins found in other crustaceans.

**Figure 6:**
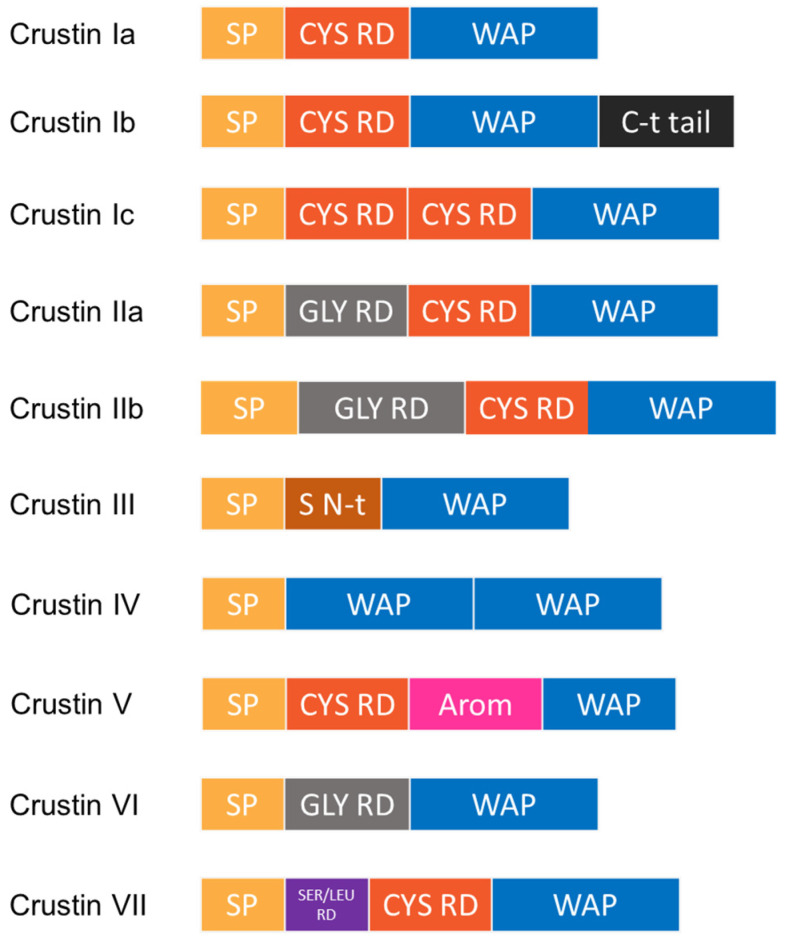
Structural features of the seven types and subtypes of crustins. Adapted from *Molecular and Functional Diversity of Crustin-Like Genes in the Shrimp Penaeus vannamei* [87]. (SP = signal peptide; CYS RD = Cys-rich domain; WAP = whey acidic protein; C-t = C-terminus; GLY RD = Gly-rich domain; S N-t = short N-terminus region; Arom = aromatic residues; SER/LEU RD = Ser/Leu-rich domain).

Several attempts have been made to classify crustins. The first three-type classification was proposed by Smith et al. [118]. Then, in 2015, a five-type classification was put forward by Tassanakajon et al. [119]. More recently, a complete classification into seven types (10 types when considering sub-classifications) on the basis of structural characteristics and phylogenetic analysis (Figure 6) was proposed by Li et al. [87]. 

Type I crustins contain a WAP domain at the carboxyl terminus and eight Cys residues; the Cys residues fold to form four disulfide bonds and create a tightly packed structure. Type Ia crustins are, in fact, the classic type I crustins, and they are, together with type II, the most widely studied type of crustins. Several mechanisms underlying the antibacterial and antiviral activities of these peptides have been reported over the years. In this regard, an interesting case of novel antibacterial activity was described by Liu et al., in 2014 [120]. These authors reported the discovery of type Ia crustin *Mj*CruI-1, finding a new function of crustins, namely in improving clearance of bacteria by enhancing the phagocytosis of blood cells. On the other hand, type Ib (MT375567; MT375568) and Ic (MT375569) crustins in *P. vannamei* were reported for the first time in 2020 by Li and coworkers [87]. Type Ib crustins contain a longer C-terminus region (more than 30 aa) than other crustins belonging to type I, whereas type Ic crustins have two linked Cys-rich domains before the WAP domain.

**Figure 7:**
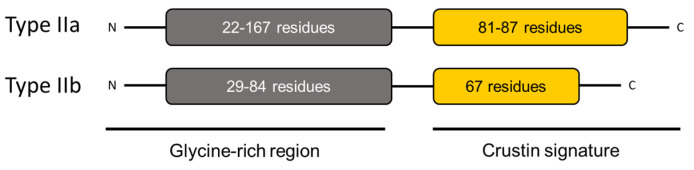
Structural differences between type IIa and IIb crustins. Adapted from [48].

Type II crustins are divided into subgroups IIa and IIb and contain a Gly-rich domain, a Cys-rich domain, and a WAP domain. Sub-type IIb has a longer Gly-rich domain and is commonly named as “crustin-like”. Type II crustins are highly relevant because they confer this shrimp immunoprotection against both Gram-negative and Gram-positive bacteria. The first full characterization of a type IIb crustin (FE049920, FE049921, and JQ824114) in *P. vannamei* was conducted by Barreto et al., in 2018 [48]. From the in silico analysis of JQ824114, FE049920, and FE049921, these authors found 75% sequence similarity with *crustin-like* molecules from *P. japonicus* and *Fi*-crustin from *P. indicus*. The structural differences between type IIa and IIb crustins is well-illustrated by the number of amino acids in the Gly-rich domain, as is illustrated in Figure 7.

Type III crustins lack both Cys-rich and Gly-rich domains, but they contain a Pro/Arg-rich region or a short amine terminus region, together with the WAP domain. Initially referred to as WAP domain (SWD)-containing proteins, type III crustins are recognized for their antibacterial activities, and by their antiviral activities. A novel type III crustin from *P. vannamei* (LvSWD3, QOL09971) was identified by Yang and coworkers in 2018 [121]. This peptide contains the typical single WAP domain and shares only 40–60% sequence identity with crustins from other shrimps, having the highest homology (60%) with *Pm*SWD3 (ACF28466) from *P. monodon*. Like other type III crustins, the role of *LvSWD3* in the immune response of *P. vannamei* is not clear.

Type IV crustins are the only type containing two WAP domains and no other Cys- or Gly-rich regions [122]. Interestingly, a decade ago, the role of these double WAP domain (DWD) proteins was not clear, particularly in the case of crustaceans. However, several examples of type IV crustins with a clear role in the host innate immunity of crustaceans have since been reported. A good example is the type IV crustin from the red swamp crayfish *Procambarus clarkia*, reported in 2017 [123]. Antimicrobial activities have also been established for both SWD and DWD peptides in recent reports, such as that published by Visetnan et al., in 2017 [124].

Recently, the elucidation of the antibacterial and anti-proteinase activities of the DWD protein (type IV crustin) *Pv*DWD1 (ABR19819) from *P. vannamei* was reported by Sakunwattana et al. [125]. That study disclosed that the expression of *PvDWD1* in this species is stimulated by acute hepatopancreatic necrosis disease (AHPND) caused by *Vibrio parahaemolyticus* (*VP_AHPND_*) infection and non-lethal heat shock (NHLS) treatment. The corresponding *rPv*DWD1 protein has antibacterial activity against both the Gram-negative *VP_AHPND_* and the Gram-positive *B. megaterium*; additionally, *rPv*DWD1 protein shows inhibitory activity against secreted proteinases from *B. subtilis* and *VP_AHPND_*. On the other hand, the anti-proteinase activity and inhibition of bacterial growth of *rPv*DWD1 is explained by a strong capacity to bind to the bacteria. Those authors thus proposed that *Pv*DWD1 plays a role in innate immunity through its anti-proteinase and antimicrobial properties. In addition to the type I and II crustins, type V have a Cys-rich region, the characteristic WAP domain, and an aromatic residue-rich region between them. Type V crustins have only been found in the ant genome so far, and there are no reports of them being found in shrimp or crustaceans. Like type I and II crustins, molecules belonging to type V have a Cys-rich domain, the characteristic WAP domain, and an aromatic residue-rich region between the two. 

Type VI crustins have a Gly-rich domain and the WAP domain at the C-terminus, and they have a close structural relationship with type IIa and IIb crustins, except that they lack the Cys-rich domain. As the new type VI classification of crustins proposed by Li [87] came out less than a year ago, the only two examples available are peptide MT375589 from *P. vannamei* and MT375594 from *F. chinensis,* determined by homology using the transcriptome database in the same work. These peptides, as well as type II and VII crustins, have a long N-terminus region compared with types I, III, and IV crustins. A third candidate to be classified as type VI is crustin A (ARB15844) from *P. vannamei,* deposited in the GenBank by Li et al., in 2017 [126]. Crustin A shares 96% sequence similarity with MT375589 and the lengths of its signal peptide, Gly-rich domain, and WAP region are very similar to those of MT375589. Finally, type VII crustins are the only group containing a Ser/Leu-rich domain, which accounts for a third of the peptide sequence. The only type VII crustin reported to date is MT375590 from *P. vannamei* [87]. 

##### Function and Role against Microorganisms

Whereas hemocytes are the main site for the expression of AMPs in shrimp [127], crustins are expressed in various organs, depending on the microorganism and the stage of the infection [128]. In fact, Barreto et al., demonstrated the highest rate of expression of crustin type IIb in the gills of *P. vannamei* [48]. Likewise, two kinds of Gly-rich crustins from *Macrobrachium nippoense* are expressed in a distinct manner. In this regard, while the highest expression of *Mn*-GlyCru1 is detected in gills, *Mn*-Gly-Cru2 is predominantly expressed in hemocytes [129]. On the other hand, *Fusarium solani* causes a reduction in crustin type IIa in circulating hemocytes of *P. vannamei* [130]. These data suggest that the crustin response could be tissue-specific, depending on the infectious pathogen involved.

The expression of crustins is determined by a specific regulatory mechanism during exposure to a microorganism. For instance, the expression of LvCruU is enhanced in *P. vannamei* by the deformed epidermal autoregulatory factor-1 (Deaf1) and transcription factor 3 (LvATF3) [113]. In the prawn *M. rosenbergii*, the dorsal homolog (MrDorsal) of the nuclear-factor Kappa B (NF-κB) family upregulates the promoter activity of MrCrustin2 and MrCrustin4 [131]. Furthermore, similar results were reported by Li et al. [126]. These researchers found that the transcription factors LvDorsal and LvRelish upregulate the promoter activity of *Lv*CrustinA. As with penaeidins, the release of crustins into plasma appears to be dependent on post-translational regulation, which is triggered by an infectious agent [48].

Crustins show broad-spectrum activity against a range of microorganisms, including *Aeromonas hydrophila* [132,133], *Edwarsiella ictaluri* [134], *E. coli* [132,135,136], *M. maritypicum* [135], *P. aeruginosa* [135,136], and *V. parahaemolyticus* [133,136], among others. The antimicrobial activities of these peptides have been demonstrated, not only for free-living organisms, but also for those in biofilms. Thus, the biofilm inhibition of Ps-cr in *P. semisulcatus* was determined against biofilms of *Bacillus thuringiensis*, *B. pumilis*, *V. parahaemolyticus*, and *V. alginolyticuls* at 40 μg/mL [137]. Similarly, Ravichandran et al., showed comparable results with a *Portunus pelagicus* crustin at 100 μg/mL against *S. aureus*, *Enterococcus faecalis*, and *P. aeruginosa* biofilms [136].

Some recently described crustins from *Scylla paramamosain* have still not been classified into previously established groups, because of their particular sequences and structural characteristics, especially their cysteine pattern, which is too different to those from the previously reported crustins [138]. Thus, these molecules could have promising activity, not only as AMPs, but as molecules with regulatory hemostatic activities.

Findings regarding the structure–function relationship of crustins continue to grow. Indeed, the new classification proposed by Li opens up the possibility of re-examining protein and gene databases to correctly identify the type of crustins found to date. For example, a simple BLAST exercise with the amino acid sequence of the type VI crustin MT375589 from *P. vannamei* gives 96% sequence identity with the entry LOC113821158 of a non-characterized epsin-like protein from the same species. Table 3 provides a list of some representatives from each crustin subfamily.

#### 3.6.3. Anti-Lipopolysaccharide Factors (ALFs)

##### Structure

**Figure 8:**
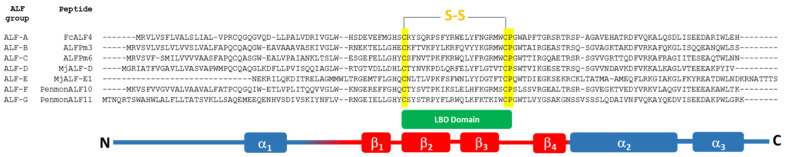
Alignment of seven representative ALFs (A-G) showing the conserved lipopolysaccharide binding domain (LBD). The secondary structure is based on the crystal structure of *ALF-Pm3* from *P. monodon*. Aromatic residues in LBD in green, cationic residues in LBD in blue, cysteine residues of disulfide loop highlighted in yellow, and highly conserved residues in red.

ALFs are by far the most widely studied AMPs in crustaceans, in part because more than 300 peptides of this class have been isolated and characterized so far. Unlike penaeidins, which are found solely in the penaeidae family, ALFs are present in crustaceans and limulids [144] (Table 4). The first ALF was isolated from the hemolymphs of the chelicerate horseshoe crabs *Limulus polyphemus* [145] and *Tachypleus tridentatus* [146]. The name ALF was assigned because they have lipopolysaccharide-binding (LPS-binding) activity, inhibiting bacterial endotoxin activation and consequently the coagulation process in limulids. The main structure signature of ALFs is the LPS binding domain (LBD), which is a highly conserved region composed of a disulfide loop formed by two Cys residues. The alignment of the seven classes of ALFs shows such structural signatures (Figure 8). 

**Figure 9:**
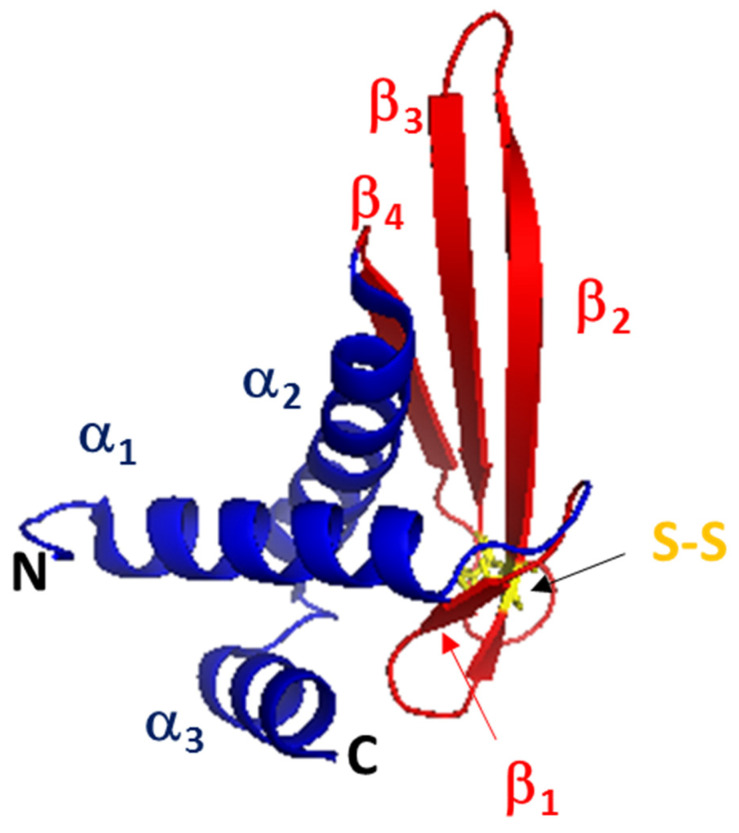
Crystal structure of *ALF-Pm3* (PDB 2JOB) from *P. monodon* showing the typical α_1_-β_1_-β_2_-β_3_-α_2_-α_3_ structure and the disulfide loop. Model built with Pymol V1.74.

Despite the large number of ALFs reported up to 2021, only one crystal structure has been described to date. In this regard, the structure of ALF-*Pm*3 from *P. monodon* was resolved by nuclear magnetic resonance (NMR) in 2009 [147], and since then, it has been taken as a model of the seven groups of ALFs. ALF-*Pm*3 contains four β-strands and three α-helices, in the order α_1_-β_1_-β_2_-β_3_-α_2_-α_3_, and the disulfide loop as part of the LBD (Figure 9).

As in the case of crustins and penaeidins, new isoforms of ALFs have progressively come to light, leading to several proposals for their classification, with that of Matos et al. [46] being widely accepted. In the case of shrimp, ALFs were divided first into two groups (A and B), and then into five (A to E). ALF-A holds both anionic and cationic polypeptides with a MW of between 11.4 and 11.5 kDa. As well as other ALFs, this group also includes the typical disulfide loop sequence (CRYSQRPSFYRWELYFNGRMWC), assigned as an LBD. In the case of *P. monodon*, several ALF isoforms have been characterized (*ALFPm1*-*5*). Of these, *ALFPm1-2* belongs to the ALF-A group, and it has been demonstrated that it is encoding by a different locus in the genome with respect to *ALFPm3-5* [148]. 

In contrast to ALF-A, the ALF-B group is formed only by highly cationic polypeptides with a MW of between 10.6 and 11.2 kDa. ALF*Pm*4 has an LPS binding sequence, CKFTVKPYLKRFQVYYKGRMWC, and codes a protein of 123 amino acid residues [148]. ALF*Pm*3 is the model most commonly used to study ALFs, because its crystal structure has been resolved using NMR (Figure 9). The alignment of ALF*Pm*3 with other representative ALFs such as ALF*Pm*6 shows that each group differs in the number of cationic amino acid residues (Arg and Lys) in the LBD (Figure 7). For example, the LBD of ALF*Pm*3 has four Lys and two Arg residues, while that of ALF*Pm*6 contains three Arg and two Lys residues, having a net charge of 6 and 5, respectively. Such a difference in cationic residues has a repercussion on the *Ip* values of these molecules (*Ip* = 9.81 for ALF*Pm*3 and *Ip* = 9.96 for ALF*Pm*6 (*Ip* = 9.96)), and the experimental results of some studies have clearly demonstrated the influence of *Ip* on antimicrobial activity [149].

The ALF-C group comprises cationic polypeptides with MWs between 11 and 11.3 kDa. An interesting example of this group is *Rsp*ALF1 from the deep-sea hydrothermal vent shrimp *Rimicaris* sp. [150]. The LBD of this peptide has a high level of sequence identity with the ALFs of several shrimps. Although the sequence results indicate the highly conserved nature of *Rsp*ALF1, it formed a new subgroup within ALF-C in shrimps. These results suggest that the unique sequence characteristics of *Rsp*ALF1, which distinguishes it from other ALFs in the phylogenetic tree, conferring functional properties associated with the deep sea that are absent in ALFs from shrimps inhabiting shallow waters.

The ALF-D group of highly anionic AMPs have MWs between 10.7 and 10.8 kDa. Recently, a new group of ALF-D molecules (*Penmon*ALF8) was found in *P. monodon* [151]. As expected, *Penmon*ALF8 is anionic, with a low isoelectric point for both the full-length peptide and LBD; it has a MW of 10.8 kDa, and it comprises a signal peptide of 26 amino acids and a mature peptide of 98 amino acids. The function of the ALF-D group was unknown until 2015 [16], when the study of *Penmon* ALF8 started to shed light on this aspect. Despite the general low level of antibacterial activity shown by reconstituted *Penmon ALF8* and its LBD, the high expression of this peptide in some tissues of *P. monodon* when challenged with *V. parahaemolyticus* accounts for its key role in the immune response of this shrimp. 

Recently, through an extensive analysis of the transcriptional profile of ALFs in *P. vannamei*, the new groups ALF-F and ALF-G were introduced. Like the other five groups, members of ALF-F and -G are encoded by distinct loci, but they conserve the same organization as the other groups [46]. This study in 2018 was the first to report the antimicrobial activity of the ALF-F group. ALF-F members share the typical α_1_-β_1_-β_2_-β_3_-α_2_-α_3_ structure and the well-conserved disulfide in LBD with the members of other ALFs. ALF-F members are cationic (theoretical *Ip*, 11.06–11.32) and they are grouped together with ALF-B and -C, due to this property.

ALF*Pm*11 from *P. monodon* was characterized two years ago [152]. This peptide, which was previously classified as an ALF-G type by Matos et al. [46], is anionic (*Ip* 5.02–6.12); however, LBD has a cationic character, as observed for the other ALF groups. The alignment of ALF*Pm*11 with ALF*Pm*3 reveals slight differences between the structures. The β_2_ strand contains two cationic residues less for ALF*Pm*11, but one more for the β_3_ strand (Figure 7, cationic residues in blue). Interestingly, ALF*Pm*3 shows a higher activity against *Acinetobacter* sp. compared to ALF*Pm*11. This observation thus suggests that slight differences in the sequence/structure of ALFs affects the selectivity of their antimicrobial properties. 

##### Function and Role against Microorganisms

In terms of antimicrobial activity, ALFs have a broad-spectrum effect against bacteria, virus, and fungi. For instance, the isoform Cf-ALF2 from *Charybdis feriatus* shows activity against Gram-positive and Gram-negative bacteria at 2.5 mM without a hemolytic or cytotoxic response at 8 mM [153]. Similarly, Li et al., identified a specific ALF, namely *Fc*ALF8, expressed in the lymphoid organ of the Chinese shrimp *F.s chinensis*, an organism that shows strong antibacterial activity against the pathogenic bacteria *V. algynolyticus*, *V. harveyi*, and *Photobacterium damselae* at MICs of 0.5–1 µM, 1–2 µM, and 1–2 µM, respectively [154]. The antibacterial activity spectrum of ALFs varies, depending on the species. For example, *Ec*ALF1 isolated from the ridge tail prawn *Exopalaemon carinicauda* shows MIC values of 2–4 µM and 16–32 µM against *V. algynolyticus* and *V. harveyi*, respectively, and 4–8 µM against *Micrococcus luteus* [155]. 

ALFs may have unspecific bactericidal activity that is not focused on microorganisms infecting crustaceans. For instance, Srisapoome et al., described the antibacterial effect of *Mr*ALF3 against the non-pathogenic Gram-positive *B. megaterium* [156]. Likewise, these compounds may have antibacterial activity against mammal bacterial pathogens. Accordingly, the ALF of the red claw crayfish *Cherax quadricarinatus* (*Cq*ALF) shows minimal bactericidal concentrations (MBCs) against *Shigella flexneri* and *Staphylococcus aureus* at <6 mM and <12 mM, respectively [157]. ALFs have shown promising antiviral and antifungal activities against yeasts and viruses, such as crab-ALF2A and crab-ALF6A purified from *Portunus trituberculatus,* showing minimal effective concentrations (MECs) of 2.11 μg/mL and 1.95 μg/mL against the yeast *Candida albicans* [158]. Additionally, Lin et al., described the disruption of the envelopes of intact WSSV virions with recombinant *rCqALF*, as well as an increased survival rate of infected animals [157].

Although many studies have been carried out to identify ALFs from neritic and freshwater crustacean species, few have focused on the immune functions of ALF molecules from deep sea animals. In this regard, Han-Jie Gu et al., characterized *Rsp*ALF1 from the deep-sea hydrothermal vent shrimp, *Rimicaris* sp. [150]. These authors demonstrated the bactericidal activity of the recombinant form of this molecule, with MIC values of between 0.25 mM and 4 mM against a broad range of Gram-negative and Gram-positive bacteria, and against yeast (i.e., *Pseudoalteromonas* sp., *Alteromonas* sp., *Bacillus* sp., *V. anguillarum*, *S. aureus*, *Micrococus luteus*, *V. harveyi*, *Pseudomonas fluorescens*, and *Streptocccus iniae*. *Yarrowia lipolytica* and *Pichia pastoris*). Overall, these findings demonstrate the wide distribution of these AMPs among crustaceans as a conserved defense mechanism against a range of microorganisms, including bacteria, fungi, and viruses.

**Table 4:** Main types of anti-lipopolysaccharide factors (ALFs) from shrimp.

ALF	Peptide Name/Code	Shrimp Species	Activity	Reference
A	*FcALF4* (JX853777)	*Penaeus chinensis*	Antiviral (WSSV)	[159]
	*ALFPm1* (EF523560)	*Penaeus monodon*	Not determined	[148]
	*ALFPm2* (EF523561)	*Penaeus monodon*	Broad spectrum antibacterial	[148]
B	*ALFPm3* (EF523559)	*Penaeus monodon*	Antifungal	[148,160]
	*ALFFc* (KJ004399)	*Penaeus chinensis*	Anti-Gram negative, antifungal	[28]
C	*ALFPm6* (AER45468)	*Penaeus monodon*	Broad spectrum antibacterial Antiviral	[161]
	*RspALF1*	*Rimicaris* sp.	Broad spectrum antibacterial Antifungal Antiviral	[150]
	*MjALF2* (AB453738)	*Penaeus japonicus*	Not determined	[162]
D	*MjALF-D* (MN416688)	*Penaeus japonicus*	Anti-Gram negative	[163]
	*PenmonALF8*	*Penaeus monodon*	Anti-Gram negative (moderate)	[151]
E	*MjALF-E1*	*Penaeus japonicus*	Anti-Gram negative	[164]
	*MjALF-E2*	*Penaeus japonicus*	Anti-Gram negative Promotes the clearance of bacteria in vivo	[164]
F	*ALFPm10* (XP_037782682)	*Penaeus monodon*	Anti-Gram positive	[46] [165]
G	*ALFPm11*	*Penaeus monodon*	Broad spectrum antibacterial Antiviral	[152]

#### 3.6.4. Stylicins

##### Structure

These anionic AMPs are composed of a Pro-rich N-terminal end and a C-terminal region containing 13 conserved Cys residues [166]. These molecules have shown a key role in the response against bacterial species in the most widely cultured marine shrimp species, namely *P. vannamei* and *P. monodon.* Thus, the stylicin family comprises two members in *P. vannamei* (Lvan-Stylicin1 and Lvan-Stylicin2), and they are highly expressed in hemocytes and in the midgut after *Vibrio* infection [166].

##### Function and Role against Microorganisms

Few studies have been devoted to the antibacterial activities of these molecules. In this regard, Sooet et al. [167] demonstrated the differential expression of 24 genes, including the overexpression of stylicin genes, after exposure to *VP_AHPND_* in *P. monodon* [167]. These data suggest a key role of these molecules in modulating the immune response in these shrimps. However, more studies are required to elucidate the mechanisms underlying the control of bacterial infection, and the possible applications of stylicins in the biotechnological field. Furthermore, a crustin purified from *P. semisulcatus* has been reported to have negligible hemolytic activity and no cytotoxic effect [137].

## 4. Marine Crustacean AMPs as Therapeutic Drugs

Bacteria have acquired extensive resistance mechanisms to protect themselves against antibiotic action. AMPs show a broad spectrum of antimicrobial activities against various microorganisms, and the diversity of modes of action involved hinder the emergence of bacterial resistance. These peptides can kill bacterial cells, either by disrupting membrane integrity, inhibiting macromolecule synthesis (proteins, DNA, and RNA), or targeting metabolic enzymes. For example, penaeidins have an amphipathic domain that targets the negatively charged bacterial membranes, and they also have a Pro-rich domain, which is determinant for their antimicrobial activity; in this case, via a non-lytic mechanism in which the Pro domain interacts with the 70S ribosome, affecting the translational process of bacteria. Cationic and anionic ALFs contain a central-β-hairpin that is able to bind to microbial surface components, and a highly hydrophobic N-terminal region that disrupts the integrity of the bacterial lipid bilayer [168]. 

Several studies have shown that AMPs exert activity against human pathogenic bacteria and fungi. Penaeidins combine the characteristics of two classes of AMP, namely Pro-rich peptides, which are active against Gram-negative bacteria, and defensins, which are active against Gram-positive bacteria. However, unlike most Pro-rich peptides, penaeidins, except for PEN5, show little activity against Gram-negative bacteria [16]. Penaedins have been reported to show activity against human pathogens *E. coli*, *K. pneumoniae*, *P. aeruginosa*, and *S. aureus* [90]. The antimicrobial activity of AMPs has also been demonstrated for biofilms. In this regard, a crustin from *P. semisulcatus* inhibits the biofilms of *B. thuringiensis* and *V. parahaemolyticus* at 40 μg/mL [137]. Similarly, Ravichandran et al., showed comparable results against *S. aureus*, *E. faecalis*, and *P. aeruginosa* biofilms with a *Portunus pelagicus* crustin at 100 μg/mL [136]. An ALF from the red claw crayfish *Cherax quadricarinatus* shows bactericidal activity against *S. flexneri* and *S. aureus.* Furthermore, ALFs have demonstrated promising activity against yeasts, as shown with crab ALF2A and crab ALF6A, purified from *Portunus trituberculatus*, against the yeast *C. albicans* [169].

Some AMPs are multifunctional molecules, performing a wide range of both immune and non-immune functions, and they are therefore considered as host defense peptides [29]. Thus, the PT-peptide, derived from the ALF of the crab *P. trituberculatus*, shows an elevated immune activity of human monocytes, mediated by an increase in reactive oxygen species (ROS) and the secretion of interferon-*γ*, interleukin-6, and tumor necrosis factor-*α* [158]. ALF-derived peptides have been shown to modulate the inflammatory response in murine macrophage cell lines [170], and they show anti-tumor activity against HeLa cells through the disruption of the cell membrane [171].

For a peptide to be considered a therapeutic drug, it needs to show not only potent antimicrobial activity, but also low toxicity to human cells. Penaedins and ALFs have potential uses in therapeutics due to a lower risk of resistance, low toxicity, and a potential role as immune modulators. In the study entitled “Discovery of synthetic penaeidin activity against antibiotic-resistant fungi”, Brando J. Cuthbertson et al. [172] conducted cytotoxicity assays to test the viability of AMPs in in vivo trials. PRD PEN4-1 and PRD PEN3-4 were tested against a human monocyte cell line (THP-1). No toxicity in either the control or treated cells was reported [172]. Similarly, Gayathri Ravichandran et al. [51] used MTT to test the effect of MrDN (AMP derived from the MrPeli-1 protein sequence) on HEK293 cells. To this end, these cells were incubated with MrDN, a potent proteasomal inhibitor (MG-132), an apoptosis inducer (positive control), and 1× PBS (negative control). No significant toxicity was observed, even at higher concentrations, whereas the positive control induced maximum toxicity in these cells [51]. Furthermore, a crustin purified from *P. semisulcatus* showed negligible hemolytic activity and no cytotoxic effect [137]. On the basis of these results, AMPs emerge as promising compounds to treat infections caused by antibiotic-resistant bacteria without causing deleterious effects to the host. A recombinant ALF peptide has been reported to show activity against *S. aureus* (MBC of 5 µM) and *E. coli* (MIC 10 µM and MBC 20 µM). The recombinant peptide was found to be non-hemolytic and non-cytotoxic in the NCI-H460 cell line at the highest concentration tested (20 µM) [173].

## 5. Antimicrobial Peptides from Chelicerates

As in crustaceans, the cellular and humoral response of chelicerates involves coagulation, phenoloxidase activation, cell agglutination, phagocytosis, and the release of antibacterial molecules [174]. Likewise, the immune response is triggered by pathogen-associated molecular patterns (PAMPs), such as LPS, β-1,3-glucans, or peptidoglycans. PAMPs are recognized through a set of PRRs [175]. The hemocytes of marine chelicerates perform immune functions via a reservoir of various defense molecules stored in granules [176]. The S and L granules of hemocytes of the Japanese horseshoe crab, *Tachypleus tridentatus*, are rich in AMPs such as tachyposin, tachycytin, tachytin, and defensins. Tachyiplesin I is among the first purified AMPs from *T. tridentatus* hemocytes. Additionally, similar AMPs, known as polyphemoine I and II, and tachyposin II, have been isolated from the hemocytes of the American horseshoe crab, *Limulus polyphemus* [177], and tachypiplesin III has also been purified from the Asian horseshoe crabs, *Caricinoscorpius rotundicauda* and *Tachypleus gigas* [178].

These peptides are cationic, with a rigid, stable, hairpin-shaped structure, where disulfide bonds join two antiparallel β-sheets. Another characteristic feature of β-hairpin peptides from horseshoe crabs is the tandem repeats of the tetrapeptide sequence; i.e., Cys, an aromatic amino acid residue, and Arg [179]. AMPs from limulids have potent and broad-spectrum antimicrobial activity against fungi, and against Gram-positive and -negative bacteria [179]. Tachyplesin I also exerts antiviral activity against human immunodeficiency virus type 1 (HIV-1) [180]. A recent study showed that tachyiplesin I and polyphemosin I have strong activity against mutants of *E. coli*, *K. pneumoniae*, and methicillin-resistant *S. aureus* [168].

The permeabilization of the bacterial membrane is the primary mechanism of the activity of tachyposin I. This peptide alters the membrane using the toroidal pore model, whereby the peptide is inserted into the lipid bilayer of the membrane perpendicularly through the formation of permeable channels. The interaction of this peptide with the polar head groups of lipids tilts and connects the lipids of the two leaflets, leading to a continuous curve through the pore. When antimicrobial actions against Gram-negative and -positive bacteria and fungi are compared, tachyposin I and polyphemusin I exert similar activities [181]. 

**Figure 10:**
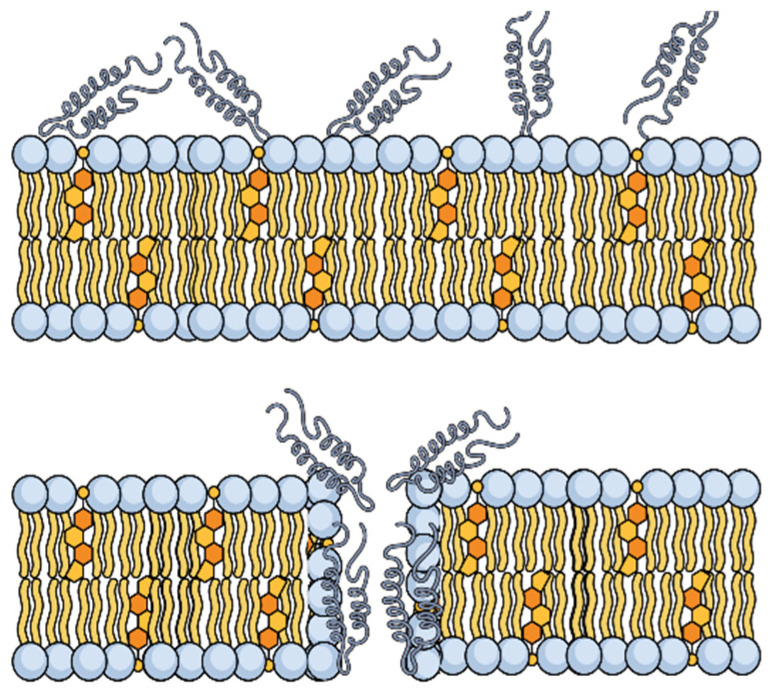
Schematic illustration of toroidal pore formation induced by the β-hairpin structures of tachyposin I.

These peptides, when in high concentrations oriented on the surface of bacteria, act like a detergent by forming transient toroidal holes. The peptide helices insert into the membrane to induce the lipid monolayers to continually fold through the pore, so that both the peptides and lipid head groups coat the water core [182] (Figure 10).

**Figure 11:**
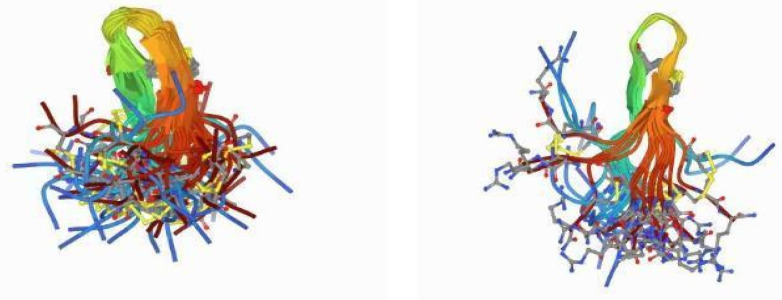
The β-hairpin structures of tachyposin I and polyphemusin I are essential for their antimicrobial activity, amphipathicity, and hydrophobicity. (Tachyplesin I 1ma2_models and Polyphemusin-1) [183].

Comparative studies of peptide families provide information on the meaning of each characteristic. Tachypiplesin I–III and polyphemosin I and II have similar structures but different Ips, net charges, hydrophobicities, and amphipathicities, which contribute to different antimicrobial activities [168] (Figure 11).

Defensin and tachycitin have been isolated from the granular hemocytes of the horseshoe crab *Tachypleus tridentatus*; both proteins also have a broad antimicrobial activity, especially against Gram-negative bacteria, and to a lesser extent, against Gram-positive bacteria and fungi [184,185].

**Figure 12:**
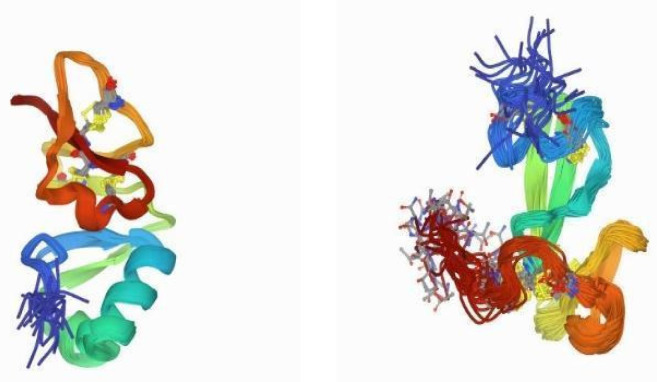
Defensin and tachycitin from the horseshoe crab *Tachypleus tridentatus*.

Defensins are hydrophobic cationic peptides with six to eight Cys residues, and two antiparallel β-sheets, with or without an α-helix. Defensins are present in invertebrates, mammals, human leukocytes, and various vertebrate epithelial cells [186]. Tachycytin has a unique structure among invertebrates, with a three-stranded β-sheet in the N-terminal region, and a two-stranded β-sheet, forming a loop in the shape of a β-hairpin, followed by a short helical turn in the C-terminal region [187] (Figure 12).

The antimicrobial activity of defensins is primarily attributed to their ability to insert into the target membrane, resulting in the permeabilization and destruction of the cell membrane. Furthermore, most defensins contain a distinct hydrophobic and hydrophilic region. The latter is rich in basic amino acids, and is essential for forming electrostatic interactions between cationic defensins and acid phospholipids in the membrane of microorganisms, and for inserting the peptides into the cell membrane [186]. The hydrophobic nature and unique structure of the N-terminal domain in large defensins suggest a new mechanism of action against Gram-positive bacteria that is not affected by salt concentration [188].

AMPs from marine arthropods mostly show high antimicrobial efficacy in vitro, but only a few of these compounds have shown efficacy in vivo. The β-hairpin peptides are highly effective against bacterial infections in animal models. For example, the in vivo efficacy of polyphemusin I against *Pseudomonas aeruginosa* in animal models was reported to be more significant than its in vitro efficacy [183].

## 6. Synthesis of Antimicrobial Peptides from Marine Arthropods

Although the experimental synthesis of peptides has experience great advances in recent years [189], the preparation of many of crustacean AMPs has not been described. To the best of our knowledge, only the groups of Cuthbertson and Bullesbach [190] have been able to prepare a few penaeidins (around 50 amino acids) using native chemical ligation (NCL) [191]. NCL is highly convenient for larger peptides containing Cys residues, such as crustacean AMPs. This technique is based on the assembly of two unprotected peptides, one with a thioester in the C-terminal and the other with a Cys residue at the N-terminal. The chemoselective attack of the thiol of the Cys residue to the thioester forms another thioester, which spontaneously rearranges through a intramolecular S,N-acyl shift, with the concomitant formation of a “native” peptide bond. 

## 7. Conclusions and Future Perspectives

Although natural products are the main source of bioactive molecules, the use of AMPs from marine arthropods is still in its infancy. In this regard, the large size of most of these AMPs, and the fact that these molecules often contain multi-disulfide bridges may have hindered research in this intriguing field. Given that the structures of AMPs from marine arthropods have not been resolved, the isolation, structural determination, and analysis of the biological activities of these families of peptides has been impeded. Additionally, this lack of knowledge has further hindered their synthesis and the development of medicinal chemistry programs, as evidenced by only a small number of syntheses described in the literature to date. 

We venture that the synthetic mapping (the synthesis of 10–15 amino acid peptides covering the whole sequence, with the overlapping of a few residues (3 to 5) on the different peptides synthesized) of these AMPs will allow for the identification of the regions responsible for the activity. Then, with the most active sequences in hand, the synthesis of analogs containing natural and non-natural amino acids, disulfides, and lactam bridges to improve activity, reduce toxicity, and increase stability, will lead to the identification of AMP candidates to treat infections caused by multi-drug-resistant bacterial strains not only in crustacean and fish (salmon, trout, etc.) aquaculture production, but also in humans. In conclusion, AMPs from marine arthropods should be added to the drug discovery toolbox needed to tackle the challenges of fighting infections.

## Figures and Tables

**Table 1:** Diversity of AMPs in Crustacea. Entries marked by an asterisk (*) in the third column were determined using the PHI-Blast alignment tool (https://blast.ncbi.nlm.nih.gov/Blast.cgi, accessed on 1 March 2021).

AMP	Characteristics	Sequence Affinity with Other Animal Taxa	Order	Family	Species	References
Bac-like	6.5 kDa, Pro-rich, cationic	Fifth iteration, mostly Pro-rich bacterial peptides of unknown functions *	Decapoda	Carcinidae	*Carcinus maenas*	[38]
Callinectin	3.7 kDa, Pro/Arg-rich, similar to arasins, cationic	No sequence similarity detected *	Decapoda	Portunidae	*Callinectes sapidus*	[39,40]
Astacidin-2	1.8 kDa, Pro/Arg-rich, cationic	Similar to arasin and arasin-like proteins from *Scylla* spp., Hyas Araneus *	Decapoda	Astacidae	*Pacifastacus leniusculus*	[41]
Armadillidin	5.3 kDa, Gly-rich, cationic	No sequence similarity detected *	Isopoda	Armadillidiidae	*Armadillidium vulgare*	[42]
Homarin (CAP-1)	4–6 kDa, putatively amphipathic α-helical, cationic	Similar to amphibian temporins	Decapoda	Nephropidae	*Homarus americanus*	[43]
Defensins	6.8–7.2 kDa, contains three disulfide bonds, cationic	Shares six putatively homologous Cys residues with mammalian β-defensins	Decapoda	Palinuridae	*Panulirus japonicus*	[44]
Anti LPS factor	7–11 kDa, contains a highly hydrophobic N-terminal region and the two Cys residues. The 3D structure of shrimp ALF consists of three α-helices and four-stranded β-sheets; the functional LPS-binding domain is a conserved cluster of positively charged residues within a β-hairpin located between two conserved Cys residues	Widely distributed in Crustacea *	Decapoda	Penaeidae	*Metapenaeus dobsoni*, *Penaeus* spp. (9)	[24,45,46]
				Atyidae	*Neocaridina heteropoda*	
				Palaemonidae	*Macrobrachium* spp. (3), *Palaemon carinicauda*	
				Nephropidae	*Homarus americanus*	
				Parastacidae	*Cherax quadricarinatus*	
				Astacidae	*Pacifastacus leniusculus*	
				Cambaridae	*Procambarus clarkii*	
				Varunidae	*Eriocheir sinensis*	
				Oregoniidae	*Cionoecetes opilio*	
				Portunidae	*Charybdis* spp. (2)*, Portunus* spp. (2), *Scylla* spp. (4)	
			Amphipoda	Hyaelellidae	*Hyalella azteca*	
				Talitridae	*Trinorchestia longiramus*	
			Isopoda	Armadillidiidae	*Armadillidium* spp. (2)	
Scygonadin	10.8 kDa, contains two Cys residues reminiscent of ALFs, anionic	Found only in *Scylla serrata* seminal fluid; shows similarities to ALFs, which might indicate a common evolutionary origin	Decapoda	Astacidae	*Scylla serrata*	[14]
*Scylla serrata* antimicrobial protein (SSAP)	11.4 kDa, anionic	A Scygonadin homolog	Decapoda	Astacidae	*Scylla serrata*	[47]
Penaeidin	4.7–7.2 kDa, cationic, N-terminal Pro/Arg-rich domain, C-terminal domain contains an amphipathic helix and two coils constrained by three disulfide bonds	Contains at least four subgroups found only in penaeidae shrimp	Decapoda	Penaeidae	*Penaeus* spp. (12) *Penaeus monoceros*	[16]
Crustin	6–22 kDa, cationic, contains whey acidic protein (WAP) domain, further characterized by Gly-rich regions and conserved Cys residues	Found throughout Crustacea, and even in some hymenopterans *	Anostraca	Artemiidae	*Artemia salina*	[16,48]
			Decapoda	Penaeidae	*Penaeus* spp. (6)	
				Atyidae	*Neocaridina heteropoda*	
				Palaemonidae	*Macrobrachium nipponense*	
				Pandalidae	*Pandalus japonicus*	
				Alvinocarididae	*Rimicaris exoculata*	
				Palinuridae	*Panulirus* spp. (2)	
				Nephropidae	*Homarus* spp. (2)	
				Parastasidae	*Cherax quadricarinatus*	
				Astacidae	*Pacifastacus leniusculus*	
				Lithodidae	*Paralithodes camtschaticus*	
				Oregoniidae	*Hyas araneus*	
				Portunidae	*Portunus pelagicus* *Scylla* spp. (3)	
Hyastatin	11.7 kDa, cationic, contains a Gly-rich region, Pro-rich domain, and Cys-rich domain	High sequence similarity with SpHyastatin	Decapoda	Oregoniidae	*Hyas araneus* *Cionoecetes opilio*	[49]
				Portunidae	*Portunus trituberculatus*	
SpHyastatin	14.1 kDa, cationic, contains Pro-rich domain and Cys-rich domain	High sequence similarity with Hyastatin, but lacking a Gly-rich region	Decapoda	Portunidae	*Scylla paramamosain*	[49]
Arasin	4.3–4.8 kDa, cationic, Pro- and Arg-rich	Similar to Astacidin-2	Decapoda	Oregoniidae	*Hyas araneus*	[50]
Stylicin	8.9 kDa, multidomain, N-terminal Pro/Arg-rich domain, C-terminal domain contains 13 Cys residues	Found only in Penaeidae.	Decapoda	Penaeidae	*Penaeus* spp. (4) *Penaeus japonicus*	[16]
Pellino-1-derived cationic antimicrobial prawn peptide	8.0 kDa, β-sheet forming, cationic	Artificial, based on bioinformatic analyses of the structure of Pellino-1.	Decapoda	Palaemonidae	*Macrobrachium rosenbergii*	[51]
Histones or histone-derived peptides	Fi-Histidin: 2.9 kDa, enriched in Arg, Ala, Gly, Leu, Ser, cationic, α-helical structure H2A: 13.2 kDa, cationic H2B: 13.5 kDa, cationic	Highly conserved with great sequence similarity; antimicrobial action might be due to sequence at the N-terminus *	Decapoda	Penaeidae	*Penaeus* spp. (2)	[52,53]
Hemocyanin-derived peptides	C-terminus: 7.9–8.3 kDa, anionic, His-rich, α-helical structure Astacin-1: 1.9 kDa, cationic Predicted AMPs: 1.5–1.8 kDa	Highly conserved with great sequence similarity *	Decapoda	Penaeidae	*Penaeus* spp. (2)	[54,55,56]
				Astacidae	*Pacifastacus leniusculus*	
				Cambaridae	*Procambarus clarkii*	
Spgly-amp	3.98 kDa, cationic, Gly-rich	Known from a single species with a hypothetical protein detected in *Portunus trituberculatus*, no sequence similarities detected in other Crustacean families *.	Decapoda	Portunidae	*Scylla paramamosain*	[57]
Scyreprocin	9.1 kDa, cationic, Lys, Ala and Ser are the most common amino acids	Known from a single species with a hypothetical protein detected in *Portunus trituberculatus*, no sequence similarities detected in other Crustacean families *.	Decapoda	Portunidae	*Scylla paramamosain*	[58]

**Table 2:** Main types of penaeidins from shrimp.

Penaeidin Class	Peptide Name/ Accession GB	Shrimp Species	Function/ Activity	Structure, pI, MW	Reference
PEN 1/2	Lv2a (Y14925)	*Litopenaeus vannamei*	Anti-Gram positiveAntifungal		[94]
	Lv2b (AF390146)	*Litopenaeus vannamei*	Not determined		[103]
	Ls2d (AY039205)	*Litopenaeus setiferus*	Not determined		[103]
PEN 3	*Mm*PEN (KF275674)	*Metapenaeus monoceros*		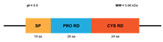	[92]
	*Fi*PEN (JX657680)	*Fenneropenaeus indicus*	Anti-Gram negative		[92]
	*Fm*PEN3 (MK092338)	*Fenneropenaeus merguiensis*	Anti-Gram negative		[91]
PEN 4	PEN4-1	*Litopenaeus schmitti*		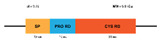	[105]
	PEN-4c (AF390149)	*Litopenaeus vannamei*			[103]
	Penaeidin 4 (QHD40386)	*Penaeus pulensis*			Unpublished results
PEN 5	*Fm*PEN5 (MK092339)	*Fenneropenaeus merguiensis*	Anti-Gram negative moderate	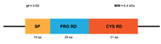	[91]
	PEN5 (FJ686018)	*Penaeus monodon*	Anti-Gram negative Anti-Gram positive Anti Fungi		[28]
PEN Family II	*Mj*Pen-II (AMH87234)	*Marsupenaeus japonicus*	Gram negative Gram positive Enhances agglutination	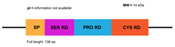	[35]

**Table 3:** Main types of crustins from shrimp.

Crustin Type	Peptide Name/ Accession GB	Shrimp Species	Function/ Activity	Structure, Ip, MW	Reference
Crustin type Ia	*MjCruI-1*	*Penaeus japonicus*	Anti-Gram negative Anti-Gram positive Phagocytosis-promoting activities	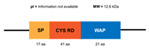	[120,139]
	Crustin type 1a-1 MT375557	*Penaeus vannamei*	Not determined		[87]
	Crustin type 1a-2 MT375558	*Penaeus vannamei*	Not determined		[87]
Crustin type Ib	*Fc*Cru Ib-1 MT375591	*Penaeus chinensis*	Not determined	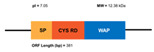	[87]
	*Fc*Cru Ib-2 MT375592	*Penaeus chinensis*	Not determined		[87]
	*Lv*Cru Ib-1 (MT375567)	*Penaeus vannamei*	Not determined		[87]
Crustin type Ic	*Fc*Cru Ic (MT375593)	*Penaeus chinensis*	Not determined		[87]
	*Lv*Cru Ic (MT375569)	*Penaeus vannamei*	Not determined		[87]
Crustin type IIa	crusFpau (EF182747)	*Penaeus paulensis*	Anti-Gram negative Anti-Gram positive	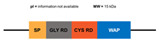	[135]
	*Lv*CrustinB	*Penaeus vannamei*	Anti-Gram negative (low) Anti-WSSV (low)		[128]
	*Re*Crustin (FJ573157)	*Rimicaris exoculata*	Anti-Gram positive		[140]
Crustin type IIb	Crustin-like *Lv* (JQ824114)	*Penaeus vannamei*	Discarded	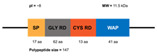	[48]
	*Lv*Cru IIb-1 (MT375581)	*Penaeus vannamei*	Not determined		[87]
	*Lv*Cru IIb-2 (MT375582)	*Penaeus vannamei*	Not determined		[87]
	*Lv*Cru IIb-3 (MT375583)	*Penaeus vannamei*	Not determined		[87]
Crustin type III	*Lv*SWD3	*Penaeus vannamei*	Antiviral(WSSV)	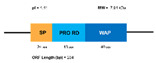	[121]
	Cru III-3 (MT375586)	*Penaeus vannamei*	Not determined		[87]
	Cru III-4 (MT375587)	*Penaeus vannamei*	Not determined		[87]
	Crustin A (ARB15844)	*Penaeus vannamei*	Anti-Gram negative		[141]
Crustin type IV	*Pv*DWD1 (ABR19819)	*Penaeus vannamei*	Anti-Gram positive, anti-proteinase	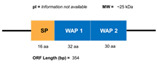	[125]
	*Pm*DWD (BI784457)	*Penaeu monodon*	Anti-proteinase (moderate)		[142]
	*Fc*DWD	*Penaeu chinensis*	Anti-Gram positive Anti-Gram negative Anti-proteinase		[143]
Crustin type VI	*Fc*CruVI (MT375594)	*Penaeus chinensis*	Not determined	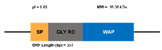	[87]
	*Lv*CruVI (MT375589)	*Penaeus vannamei*	Not determined		[87]
Crustin type VII	*Lv*CruVII (MT375590)	*Penaeus vannamei*	Not determined	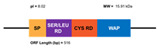	[87]
